# Endoscopic-guided decompression via modified lavage tube: salvage of life-threatening gastric outlet obstruction in a thoracic stomach

**DOI:** 10.1055/a-2687-6540

**Published:** 2025-09-05

**Authors:** Xia Wu, Sheng Zhang, Quan Wen, Faming Zhang, Bota Cui

**Affiliations:** 1 Department of Microbiota Medicine and Medical Center for Digestive Diseases, The Second Affiliated Hospital of Nanjing Medical University, Nanjing, China; 2 Key Lab of Holistic Integrative Enterology, The Second Affiliated Hospital of Nanjing Medical University, Nanjing, China

A 74-year-old woman with a history of esophagectomy/intrathoracic stomach (15 years prior) and recent retroperitoneal tumor resection (3 months prior) presented with shock, respiratory failure, and massive abdominal distension. Computed tomography (CT) revealed severe dilation of the intrathoracic stomach, which contained voluminous food debris/bezoars, causing near-total right lung collapse and apparent gastric outlet obstruction. Physical examination exhibited altered mental status, malnutrition (body mass index 16.4 kg/m ^2^ ), tachycardia, tachypnea, and hypotension despite administration of 30 mL/h of metaraminol. Standard decompression, prokinetics, and conventional endoscopy failed due to complete luminal occlusion and aspiration risk 1 . Hemodynamic instability persisted despite vasopressors.

Under general anesthesia with endotracheal intubation, a novel endoscopic decompression technique was employed. A modified 36-Fr gastric lavage tube (open-ended tip and enlarged side ports) was inserted over an ultrathin gastroscope into the esophagus. After scope withdrawal, the tube was connected to negative-pressure aspirator for high-volume suction. The ultrathin scope was reinserted alongside the tube, enabling real-time endoscopic visualization. Pulsed saline irrigation mobilized the debris; tenacious bezoars were mechanically fragmented using a lithotripsy basket via a standard gastroscope under direct vision. Following evacuation of ~1800 mL of contents, definitive endoscopy revealed a patent pylorus/duodenum, confirming functional obstruction secondary to massive retention-induced pyloric distortion within the compromised thoracic anatomy. A transendoscopic enteral tube was placed into the duodenum for immediate enteral nutrition.

The patient exhibited rapid clinical improvement: respiratory distress and shock resolved, and mental status normalized. Follow-up CT confirmed significant decompression ( Fig. 1 , Fig. 2 , Fig. 3 , Fig. 4 , Fig. 5 , Video 1 ). She was discharged with a structured plan for graded transition from enteral to oral nutrition 2 .

**Fig. 1:**
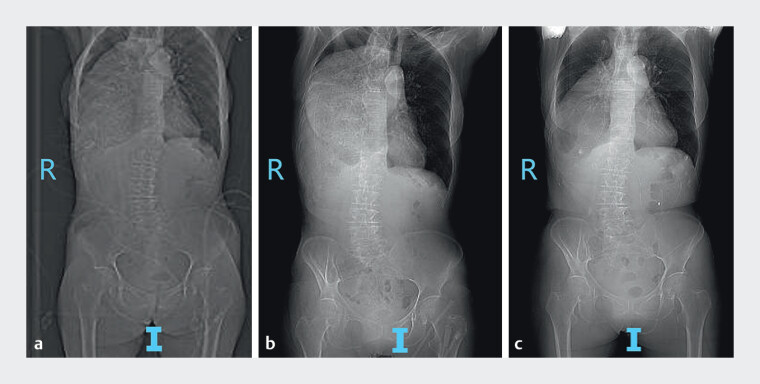
Plain radiographs of the chest and abdomen. **a** Before treatment. **b** After conservative therapy. **c** After novel endoscopic decompression. A significant reduction in the volume of gastric contents was observed after the endoscopic intervention compared with before treatment and after conservative management. R, right; I, inferior.

**Fig. 2:**
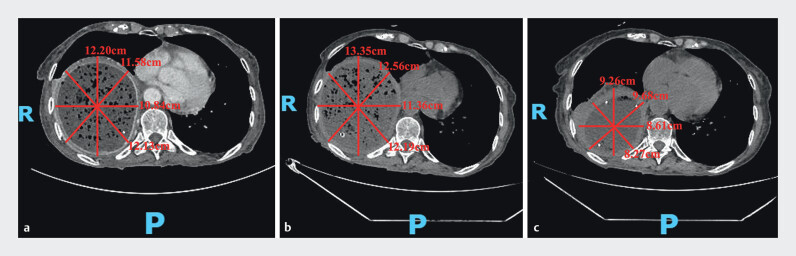
Computed tomography images obtained at the same cross-sectional level. **a** Before treatment, showing severe dilation of the right thoracic stomach, with voluminous content retention and compressive atelectasis of the right lung. **b** After conservative therapy, showing persistent massive gastric retention with minimal volume change. **c** After novel endoscopic decompression, showing marked reduction in gastric luminal distension and content volume. Quantitative assessment using the four representative lines confirmed a substantial decrease in volume compared with images from before treatment and after conservative therapy. R, right; P, posterior.

**Fig. 3:**
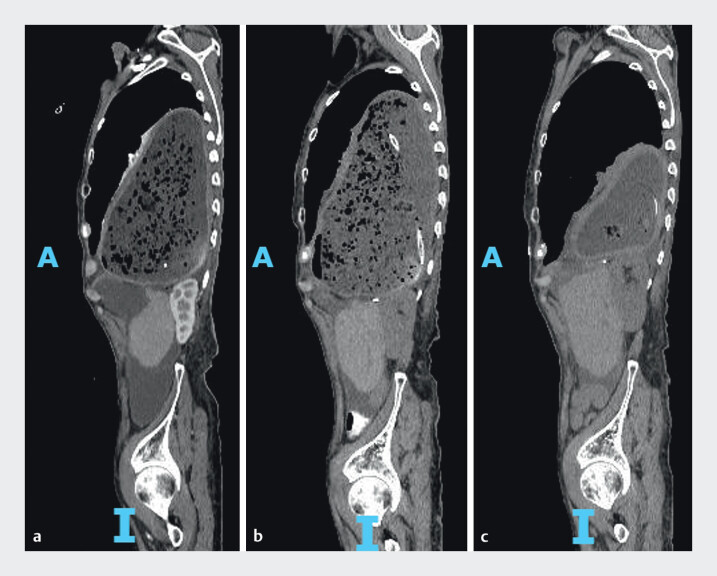
Computed tomography images obtained at the same sagittal plane level. **a** Before treatment. **b** After conservative therapy. **c** After novel endoscopic decompression. A, anterior; I, inferior.

**Fig. 4:**
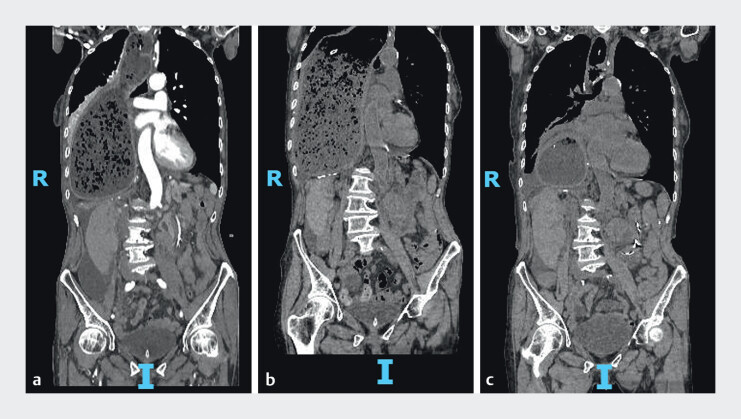
Computed tomography images obtained at the same coronal level. **a** Before treatment. **b** After conservative therapy. **c** After novel endoscopic decompression. R, right; I, inferior.

**Fig. 5:**
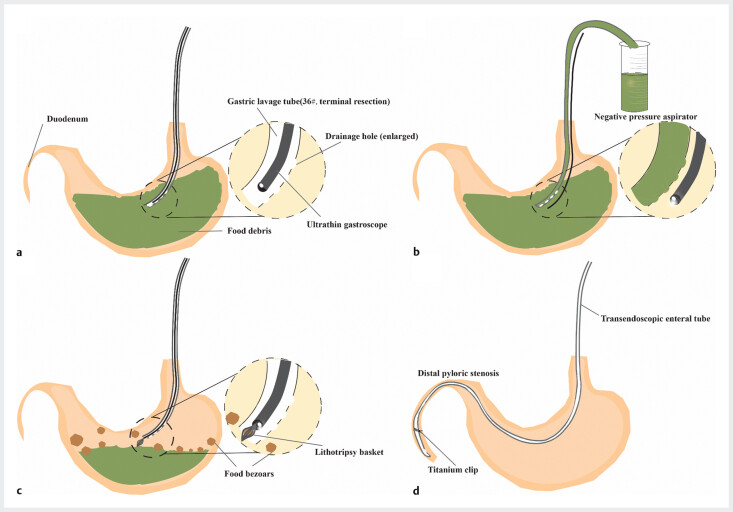
Schematic of real-time endoscopic-guided decompression using a modified gastric lavage tube for the treatment of life-threatening intrathoracic gastric retention. **a** An ultrathin gastroscope was inserted through the lumen of a modified 36-Fr gastric lavage tube (open-ended tip and enlarged side holes), and guided the placement of the tube into the thoracic stomach. **b** After scope withdrawal, the tube remained in place and was connected to a negative-pressure aspirator for high-volume suction. The position of the gastric lavage tube was adjusted under the direct vision of the endoscope to ensure smooth suction. **c** Large, tenacious bezoars were mechanically fragmented using a lithotripsy basket via the gastroscope; then, water pulse irrigation was performed to suspend the crushed debris and facilitate subsequent suction. **d** After removal of massive gastric contents (~1800 mL), the endoscope successfully reached the pylorus, and mid-gut transendoscopic enteral tube was placed for enteral nutrition.

Successful management of life-threatening gastric outlet obstruction in a thoracic stomach using endoscopic-guided decompression via modified lavage tube.Video 1

This critically complex case demonstrates a pioneering real-time endoscopic-guided decompression technique using a modified lavage tube ( Video 1 , Fig. 5 ). The approach overcame complete luminal obstruction from massive bezoars in distorted anatomy, enabled definitive diagnosis of functional obstruction, established urgent enteral access, and circumvented high-risk surgery, providing a life-saving minimally invasive salvage strategy.

Endoscopy_UCTN_Code_TTT_1AO_2AL

## Notes

Endoscopy E-Videos https://eref.thieme.de/e-videos
*E-Videos* is an open access online section of the journal *Endoscopy* , reporting on interesting cases and new techniques in gastroenterological endoscopy. All papers include a high-quality video and are published with a Creative Commons CC-BY license. Endoscopy E-Videos qualify for HINARI discounts and waivers and eligibility is automatically checked during the submission process. We grant 100% waivers to articles whose corresponding authors are based in Group A countries and 50% waivers to those who are based in Group B countries as classified by Research4Life (see: https://www.research4life.org/access/eligibility/ ). This section has its own submission website at https://mc.manuscriptcentral.com/e-videos .
